# Supplementary material for: CXCR5+PD-1+ follicular helper CD8 T cells control B cell tolerance
Source: Nat Commun. 2019 Sep 27;10:4415. doi: 10.1038/s41467-019-12446-5 (PMC6765049; doi:10.1038/s41467-019-12446-5)
Supplement: Supplementary file 1 — Supplementary Information [file 41467_2019_12446_MOESM1_ESM.pdf]

Supplementary Information

CXCR5^{+}PD-1^{+} follicular helper CD8 T cells control B cell tolerance

Chen et al.

Contents:

Supplementary Figures 1-24

Supplementary Tables 1-3

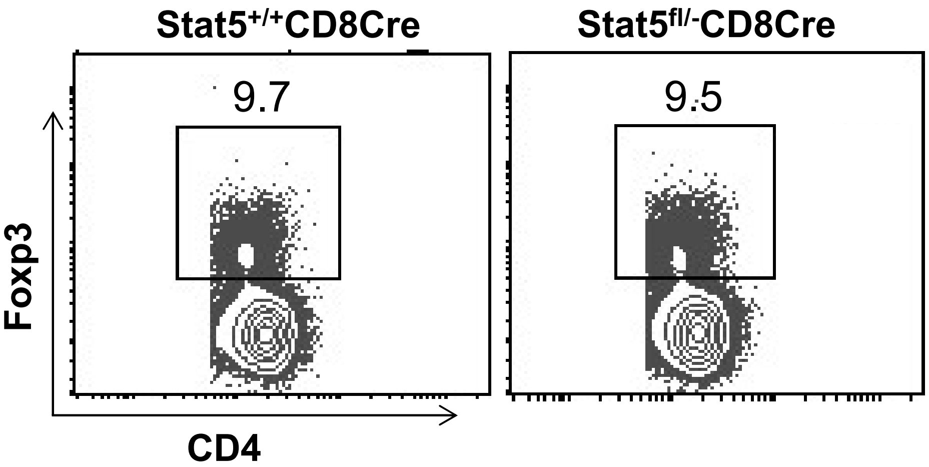

Supplementary Figure 1. Stat5 deletion in CD8^{+} T cells has no effect on CD4 Treg cell development. The splenocytes from Stat5$^{+/+}$CD8Cre or Stat5$^{fl/-}$CD8Cre mice were stained with anti-CD4 and anti-CD8 antibodies, followed by Foxp3 intracellular staining. Numbers indicate percentages of Foxp3^{+} cells in the gated CD4^{+} population. Data shown are representative of 3 independent experiments.

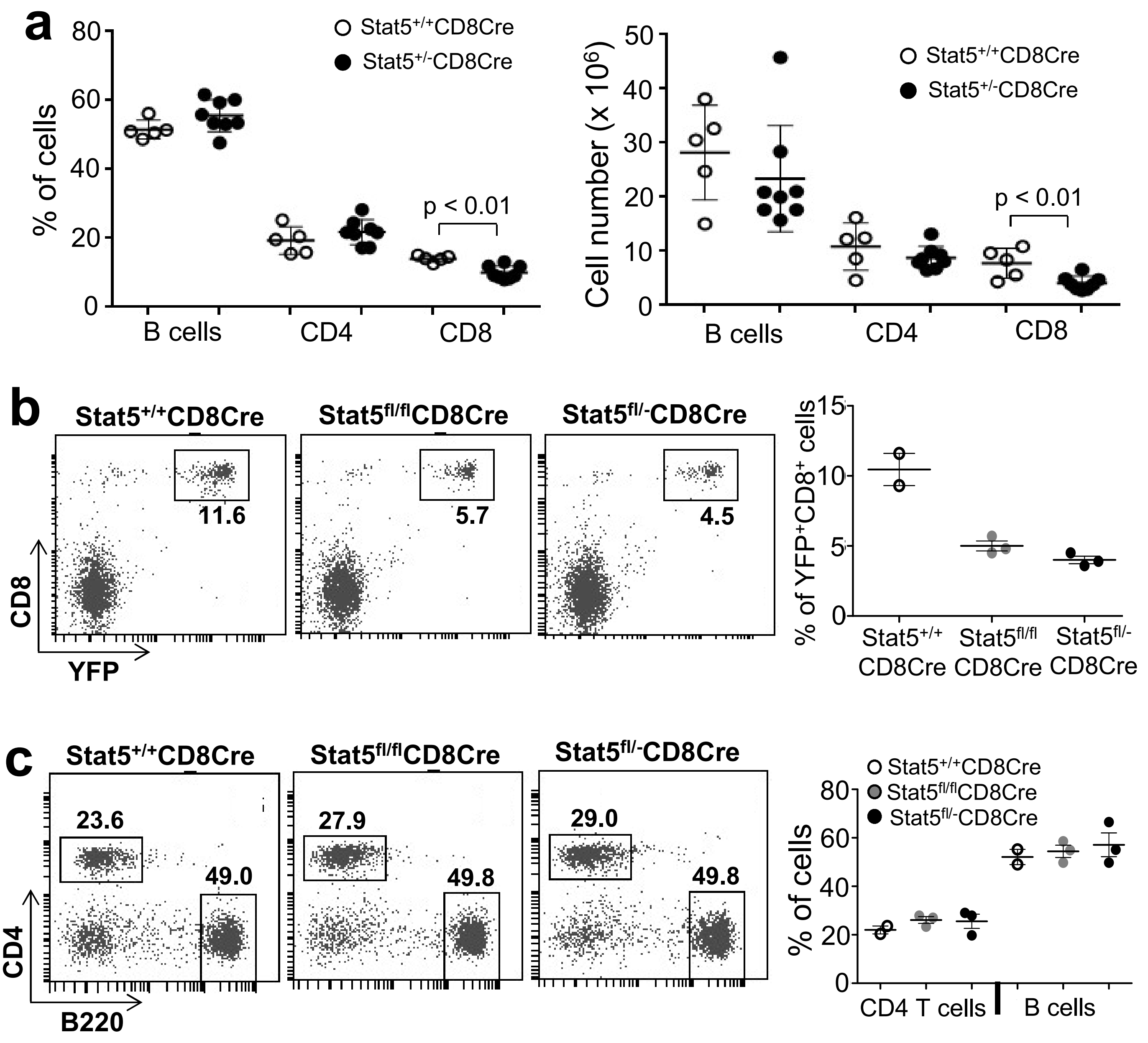

Supplementary Figure 2. Lymphocyte development in Stat5$^{+/+}$CD8Cre/YFP, Stat5$^{+/-}$CD8Cre/YFP, Stat5$^{fl/fl}$CD8Cre/YFP and Stat5$^{fl/-}$CD8Cre/YFP mice. a Lymphocyte development in Stat5$^{+/-}$CD8Cre/YFP mice. The percentages and numbers of splenic B cells, CD4^{+} and CD8^{+} T cells in Stat5$^{+/+}$CD8Cre/YFP and Stat5$^{+/-}$CD8Cre/YFP mice are shown. Data are obtained from 5 Stat5$^{+/+}$CD8Cre/YFP and 8 Stat5$^{+/-}$CD8Cre/YFP mice. **b,c** Lymphocyte development in Stat5$^{fl/fl}$CD8Cre/YFP and Stat5$^{fl/-}$CD8Cre/YFP mice. The percentages and numbers of splenic YFP^{+}CD8^{+} T cells, B cells, and CD4^{+} T cells in Stat5$^{fl/fl}$CD8Cre/YFP and Stat5$^{fl/-}$CD8Cre/YFP mice. Mean ± SD is shown. P-values were calculated with the unpaired two-tailed Student's *t*-test. Data are representative of or obtained from 2 Stat5$^{+/+}$CD8Cre/YFP, 3 Stat5$^{fl/fl}$CD8Cre/YFP and 3 Stat5$^{fl/-}$CD8Cre/YFP mice.

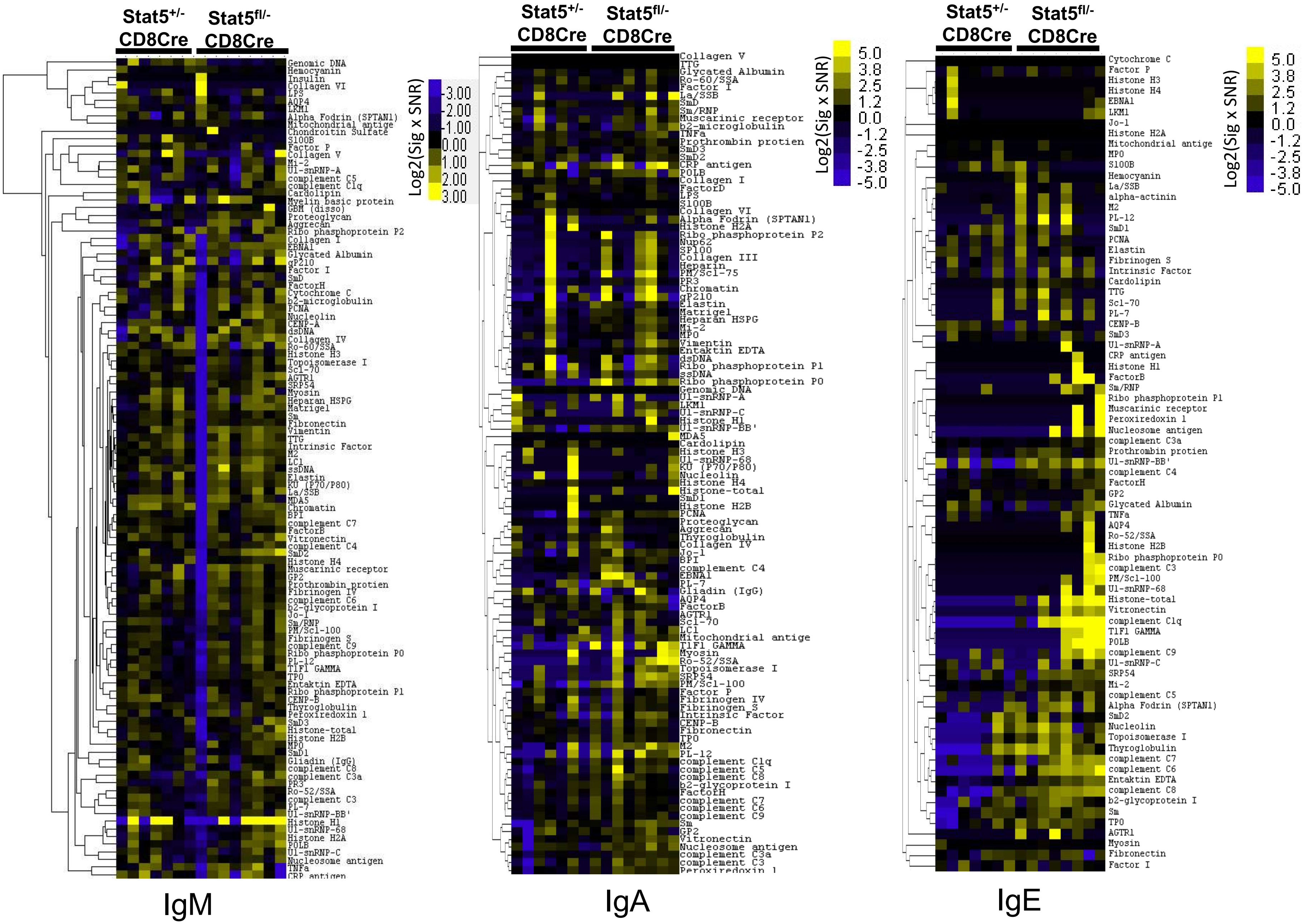

Supplementary Figure 3. **IgM , IgA and IgE autoantibody production in Stat5$^{fl/-}$CD8Cre mice.** The sera from 2 month-old Stat5$^{+/-}$CD8Cre or Stat5$^{fl/-}$CD8Cre mice were screened by the autoantigen array.

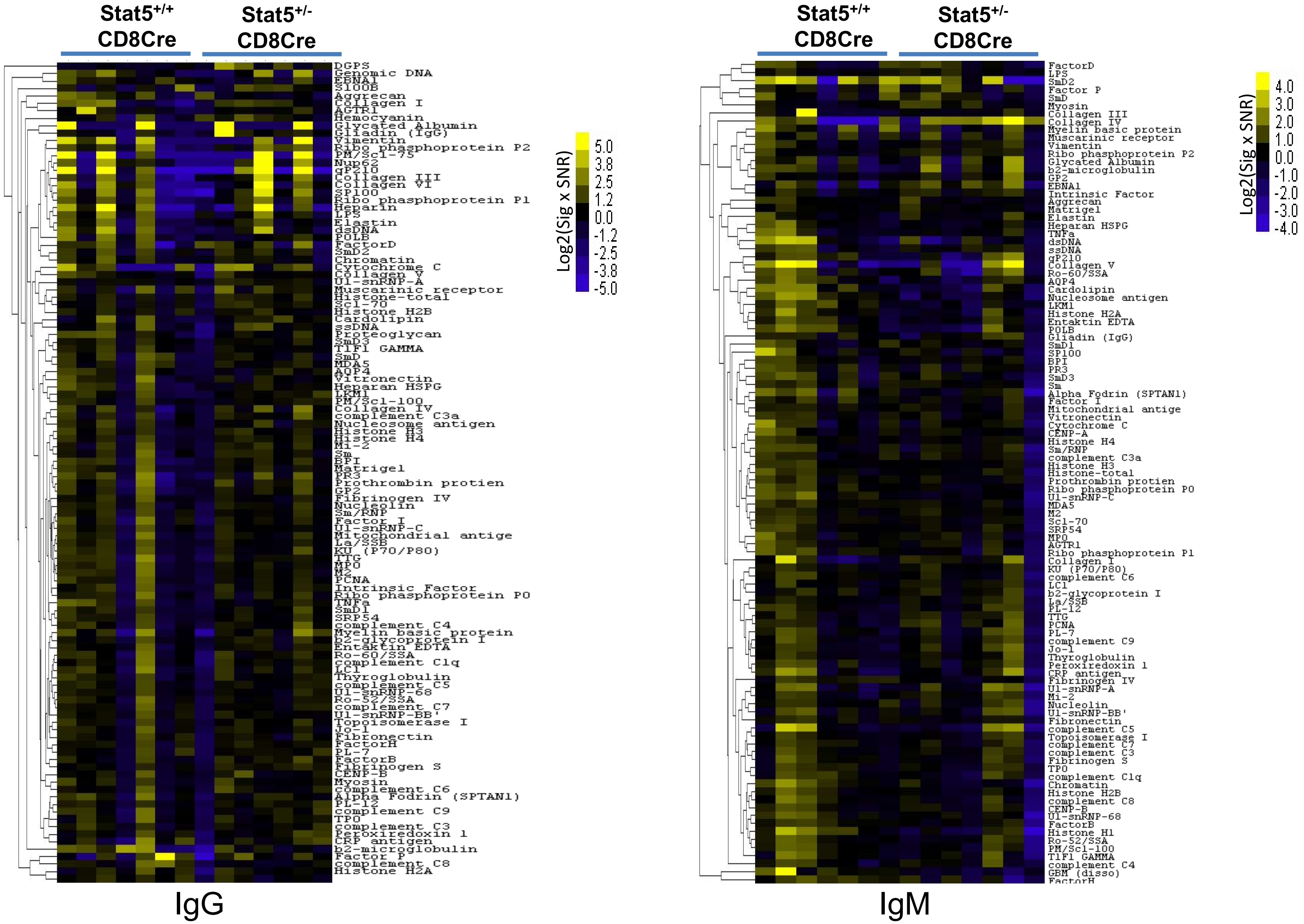

Supplementary Figure 4. IgG and IgM autoantibody production in Stat5$^{+/+}$CD8Cre and Stat5$^{+/-}$CD8Cre mice. The sera from 2 month-old Stat5$^{+/+}$CD8Cre or Stat5$^{+/-}$CD8Cre mice were screened by the autoantigen array.

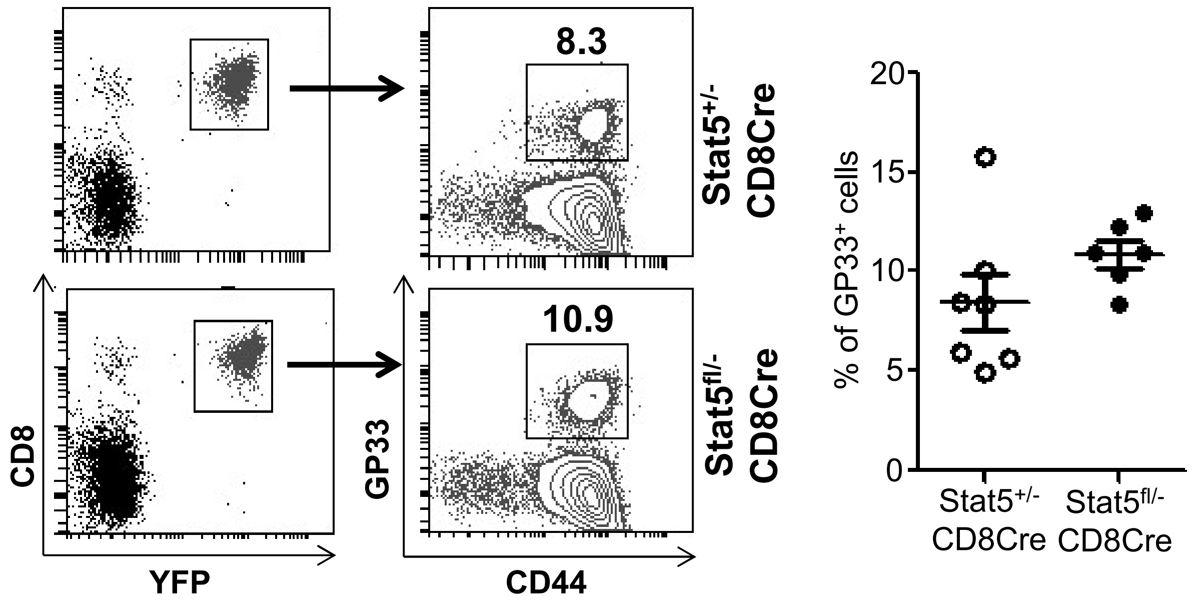

Supplementary Figure 5. Stat5-deficient CD8$^+$ T cells respond normally to LCMV infection. Stat5$^{+/-}$CD8Cre and Stat5$^{fl/-}$CD8Cre mice were infected with LCMV (Armstrong). Eight days after infection, splenocytes were stained with anti-CD8 and anti-CD44 antibodies, and GP33-Tetramer. Numbers indicate percentages of CD8$^+$YFP$^+$GP33$^+$ cells in the gated CD8$^+$YFP$^+$ population (left) and each dot represents an individual mouse (right). Mean ± SD is shown. P-values were calculated with the unpaired two-tailed Student's *t*-test. Data shown are obtained from 7 Stat5$^{+/-}$CD8Cre/YFP and 6 Stat5$^{fl/-}$CD8Cre/YFP mice.

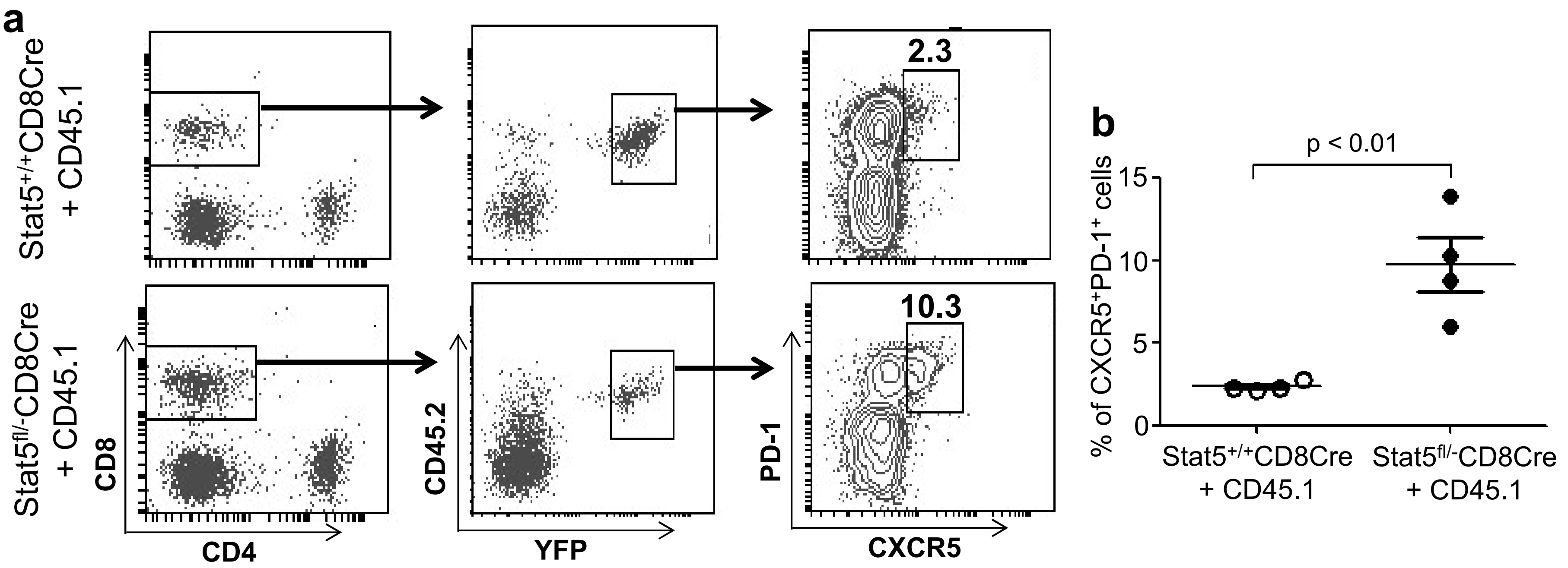

Supplementary Figure 6. Markedly increased Stat5-deficient CXCR5^{+}PD-1^{+}CD8^{+} T cells in BM chimeric mice following acute LCMV infection. BM cells from Stat5$^{+/+}$CD8Cre/YFP or Stat5$^{fl/-}$CD8Cre/YFP mice were mixed with BM cells from wild-type CD45.1 congenic mice at 1:1 ratio and transplanted into Rag1-deficient mice. Eight weeks after transplantation, the recipients were infected with LCMV. Eight days after infection, splenocytes were stained with anti-CD45.2, anti-CD4, anti-CD8, anti-CXCR5 and anti-PD-1 antibodies. Numbers indicate percentages of CXCR5^{+}PD-1^{+} cells in the gated CD45.2^{+}YFP^{+}CD8^{+} population (**a**) and each dot represents an individual mouse (**b**). Mean ± SD is shown. P-values were calculated with the unpaired two-tailed Student's *t*-test. Data shown are obtained from 4 Stat5$^{+/+}$CD8Cre/YFP and 4 Stat5$^{fl/-}$CD8Cre/YFP mice.

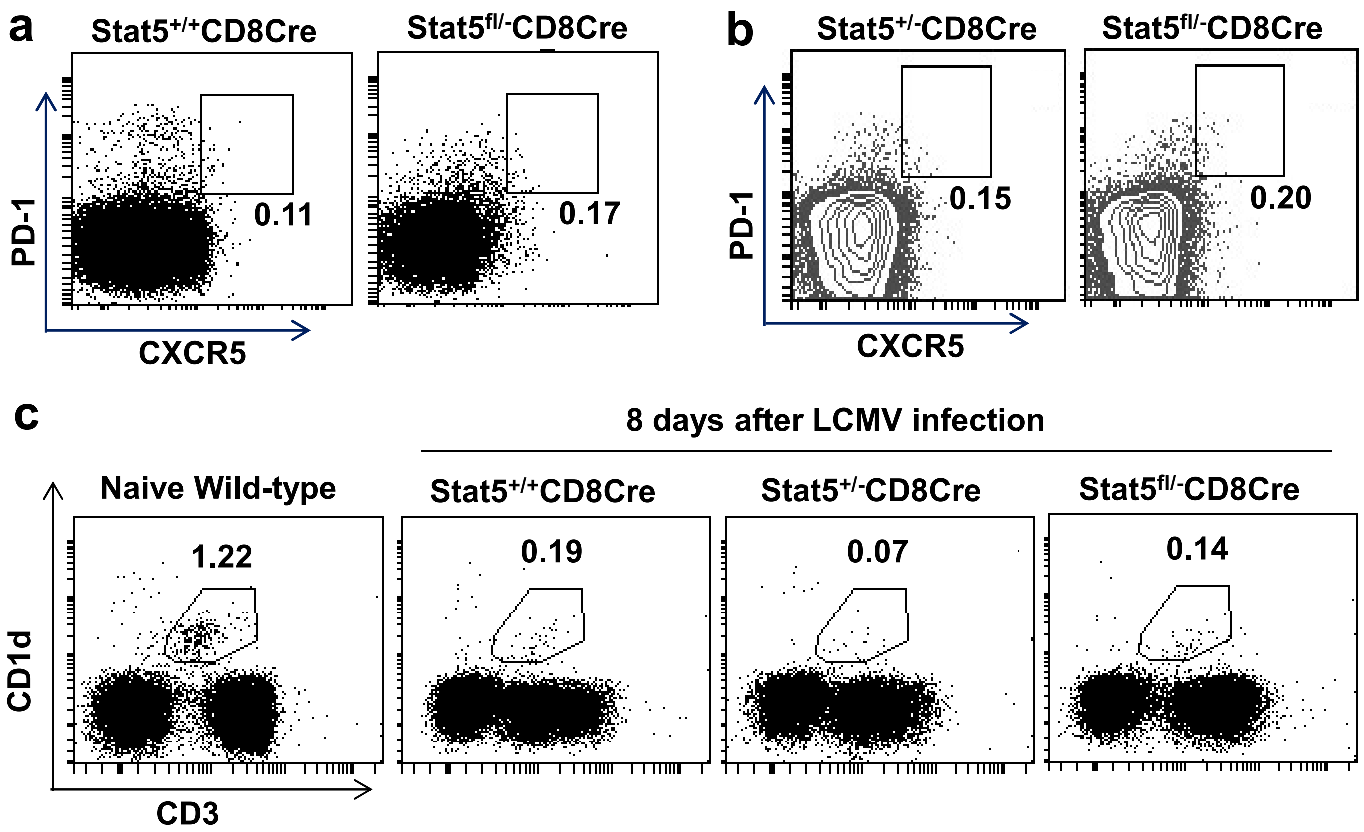

Supplementary Figure 7. Few CXCR5$^+$PD-1$^+$CD8$^+$ T cells in naïve Stat5$^{fl/-}$ CD8Cre and control mice, and disappearance of NKT cells in mice following acute LCMV infection . Splenocytes from naive Stat5$^{+/+}$ CD8Cre and Stat5$^{fl/-}$ CD8Cre mice **(a)** or naive Stat5$^{+/-}$ CD8Cre and Stat5$^{fl/-}$CD8Cre mice **(b)** were stained with anti-CD4, anti-CD8, anti-CXCR5 and anti-PD1 antibodies. Numbers indicate percentages of CXCR5$^+$ PD-1$^+$ cells in the gated YFP$^+$CD8$^+$ population. **c** Splenocytes from naïve wild-type or LCMV-infected Stat5$^{+/+}$CD8Cre, Stat5$^{+/-}$CD8Cre or Stat5$^{fl/-}$CD8Cre mice were stained with anti-CD3 antibody and CD1d-tetramer. Numbers indicate percentages of CD3$^+$CD1d$^+$ cells in live splenocytes. Data showed are representative of 8 (**a**) or 6 (**b**) pairs of mice or 3 (**c**) independent experiments.

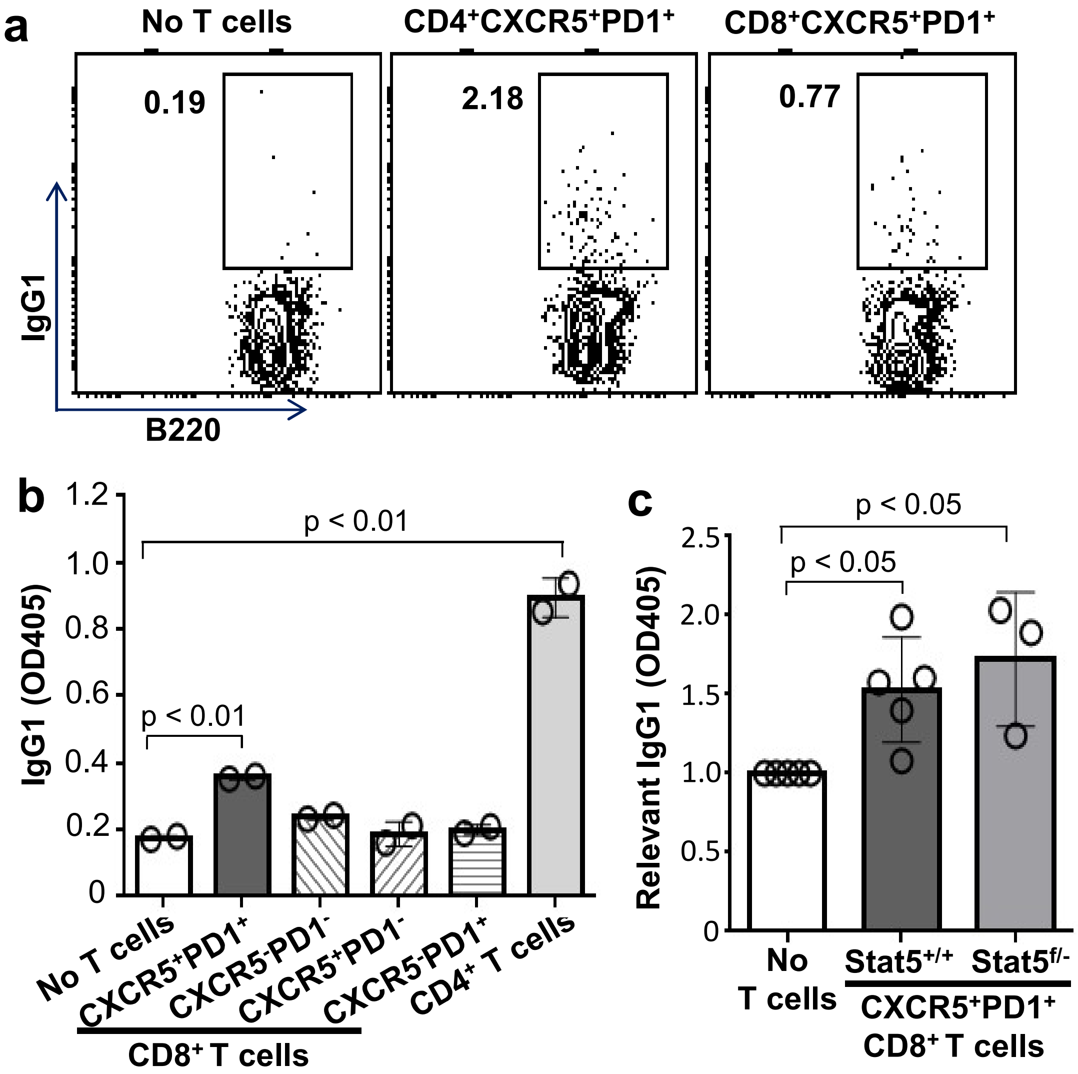

Supplementary Figure 8. Direct support of B cells by $CXCR5^+PD\text{-}1^+CD8^+$ T cells to produce antibodies in vitro. a-c B cells and $CXCR5^+PD\text{-}1^+CD4^+$ and $CXCR5^+PD\text{-}1^+CD8^+$ T cells were sorted from wild-type mice 8 days after acute LCMV infection. B cells were cultured with or without indicated $CD8^+$ T cells. **a** Numbers indicate percentages of $IgG1^+$ cells in the gated $B220^+$ population. Bar graphs show the levels of IgG1 in the supernatant of B cells co-cultured with the indicated T cell subpopulation (**b**), or $Stat5^{+/+}CXCR5^+PD\text{-}1^+CD4^+$ or $Stat5^{-/-}$ $CXCR5^+PD\text{-}1^+CD8^+$ T cells (**c**). Mean ± SD is shown. P-values were calculated with the unpaired two-tailed Student's *t*-test. Data showed are representative of 3 (**a**), 5 (**b**) or 4 (**c**) independent experiments.

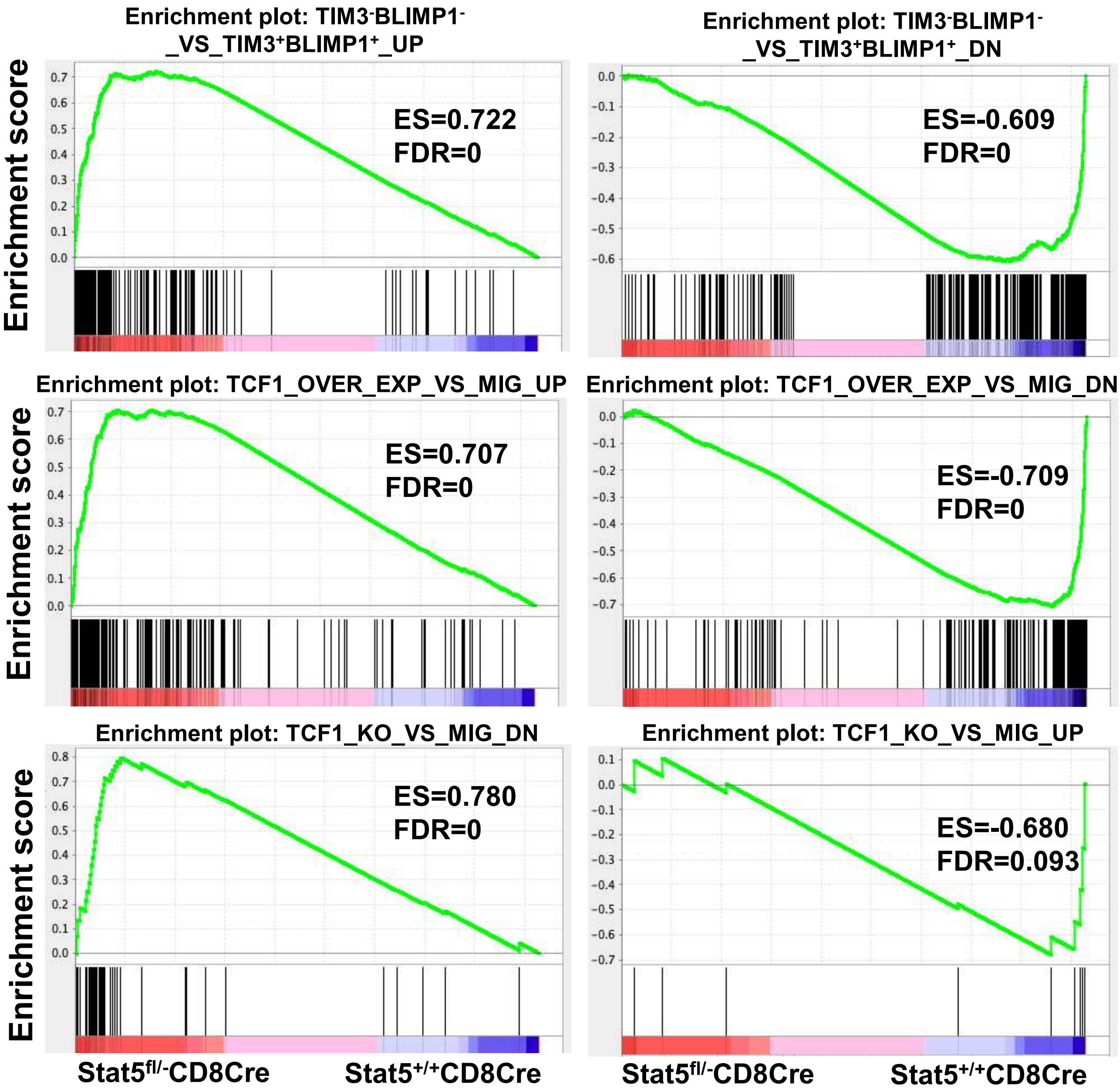

Supplementary Figure 9. Comparative GSEA of the Tfh signatures in wild-type and Stat5-deficient CXCR5$^+$PD-1$^+$CD8$^+$ T cells. GSEA of the Tfh-like signatures (GEO accession code GSE85367) in CXCR5$^+$PD-1$^+$CD8$^+$ T cells from Stat5$^{fl/-}$CD8Cre/YFP and Stat5$^{+/+}$CD8Cre/YFP mice.

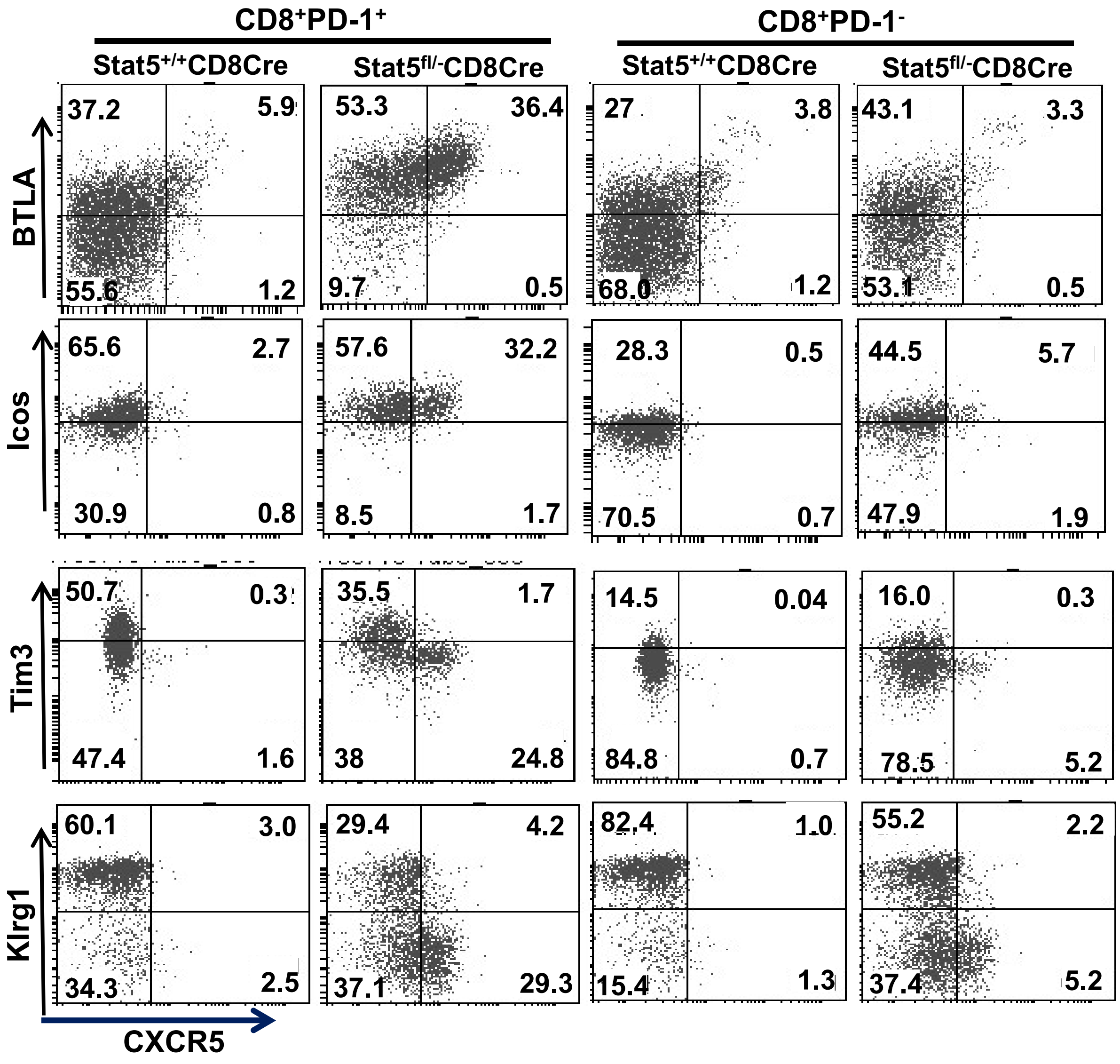

Supplementary Figure 10. Expression of Tfh-related and other genes in wild-type and Stat5-deficient CXCR5$^+$PD-1$^+$CD8$^+$ and CXCR5$^+$PD-1$^-$CD8$^+$ cells. Stat5$^{+/+}$CD8Cre and Stat5$^{fl/-}$CD8Cre mice were infected with LCMV. Eight days after infection, splenocytes were stained with the combination of anti-CD4/CD8/CXCR5/PD-1 and the indicated antibodies. Numbers in the quadrants indicate percentages of cells in the gated CD8$^+$PD-1$^+$ or CD8$^+$PD-1$^-$CD8$^+$ population. Data shown are representative of 3 pairs of Stat5$^{+/+}$CD8Cre and Stat5$^{fl/-}$CD8Cre mice.

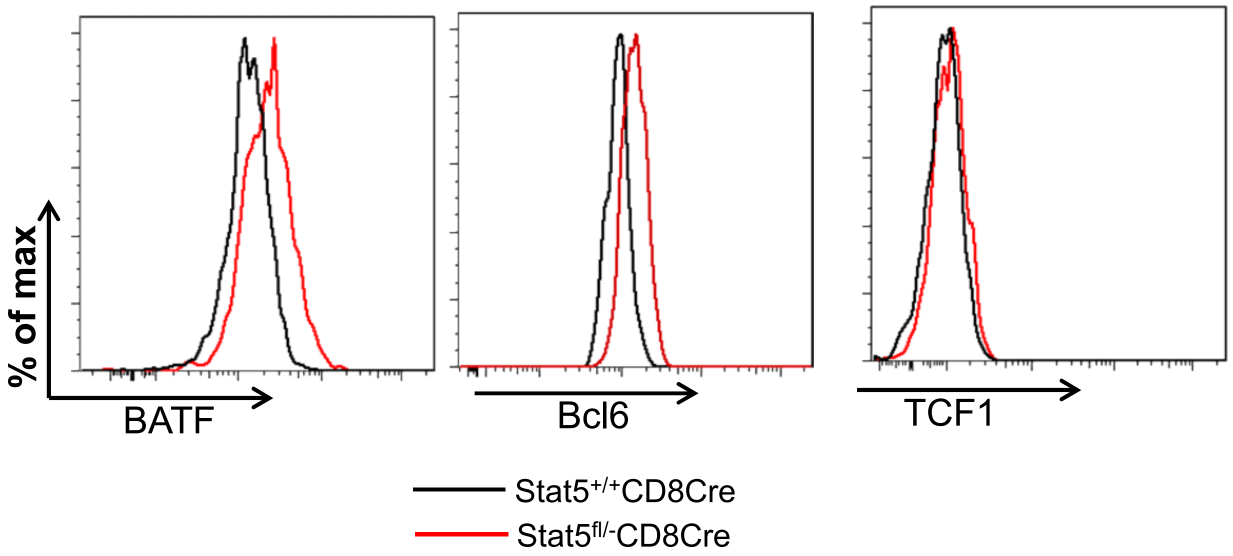

Supplementary Figure 11. Stat5 negatively regulates the expression of BATF and Bcl6, but not TCF1, in $CD8^+CXCR5^+PD\text{-}1^+$ T cells. $Stat5^{+/+}$ CD8Cre and $Stat5^{fl/-}$CD8Cre mice were infected with LCMV. Eight days after infection, splenocytes were stained with anti-CD4/CD8/CXCR5/PD-1 antibodies, followed by intracellular staining with anti-BATF, anti-Bcl6 or anti-TCF1 antibodies. Histograms show BATF, Bcl6 and TCF expression on the gated $CD8^+CXCR5^+PD\text{-}1^+$ populations. Data shown are representative of 5 experiments for Bcl6 and 3 experiments for BATF and TCF1.

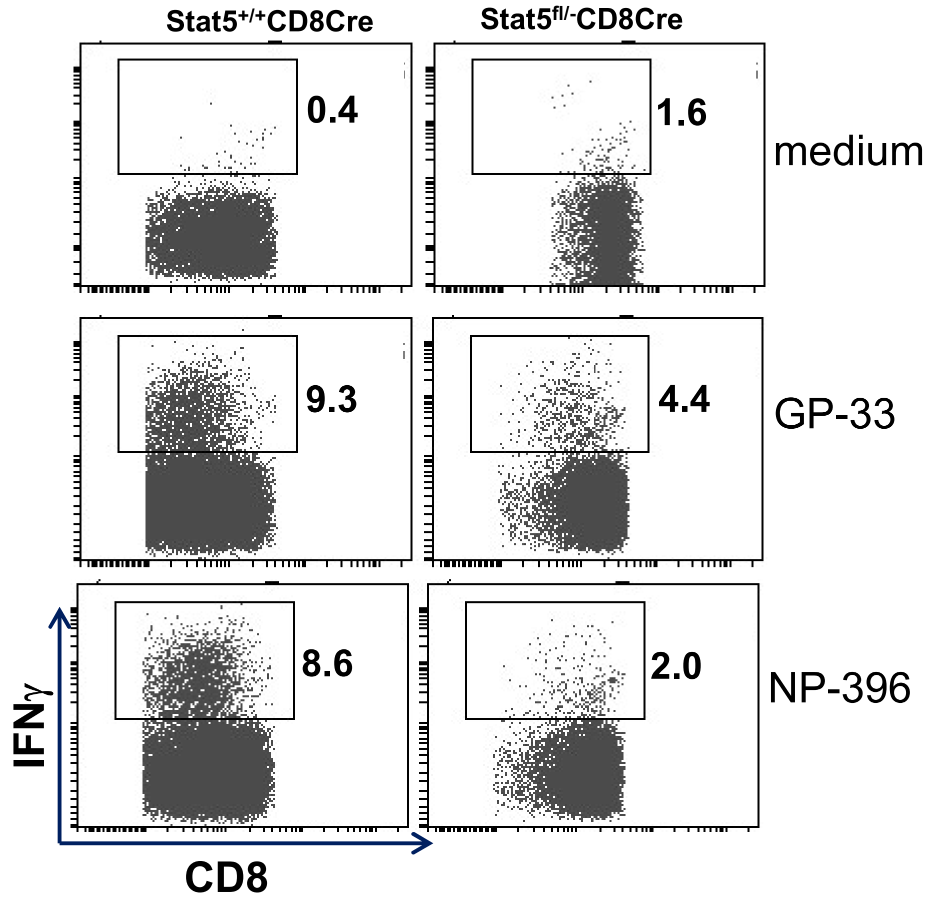

Supplementary Figure 12. Stat5 is important for IFNγ production in $CD8^+$ T cells. $Stat5^{+/+}$CD8Cre and $Stat5^{fl/-}$CD8Cre mice were infected with LCMV. Eight days after infection, splenocytes were stimulated with GP33 or NP396 peptides for 5 hours and stained with anti-CD4 and anti-CD8 antibodies, followed by intracellular staining with anti-IFNγ antibodies. Numbers indicate percentages of $IFN\gamma^+$ cells in the gated YFP^+CD8^+ population**.** Data shown are representative of 2 independent experiments.

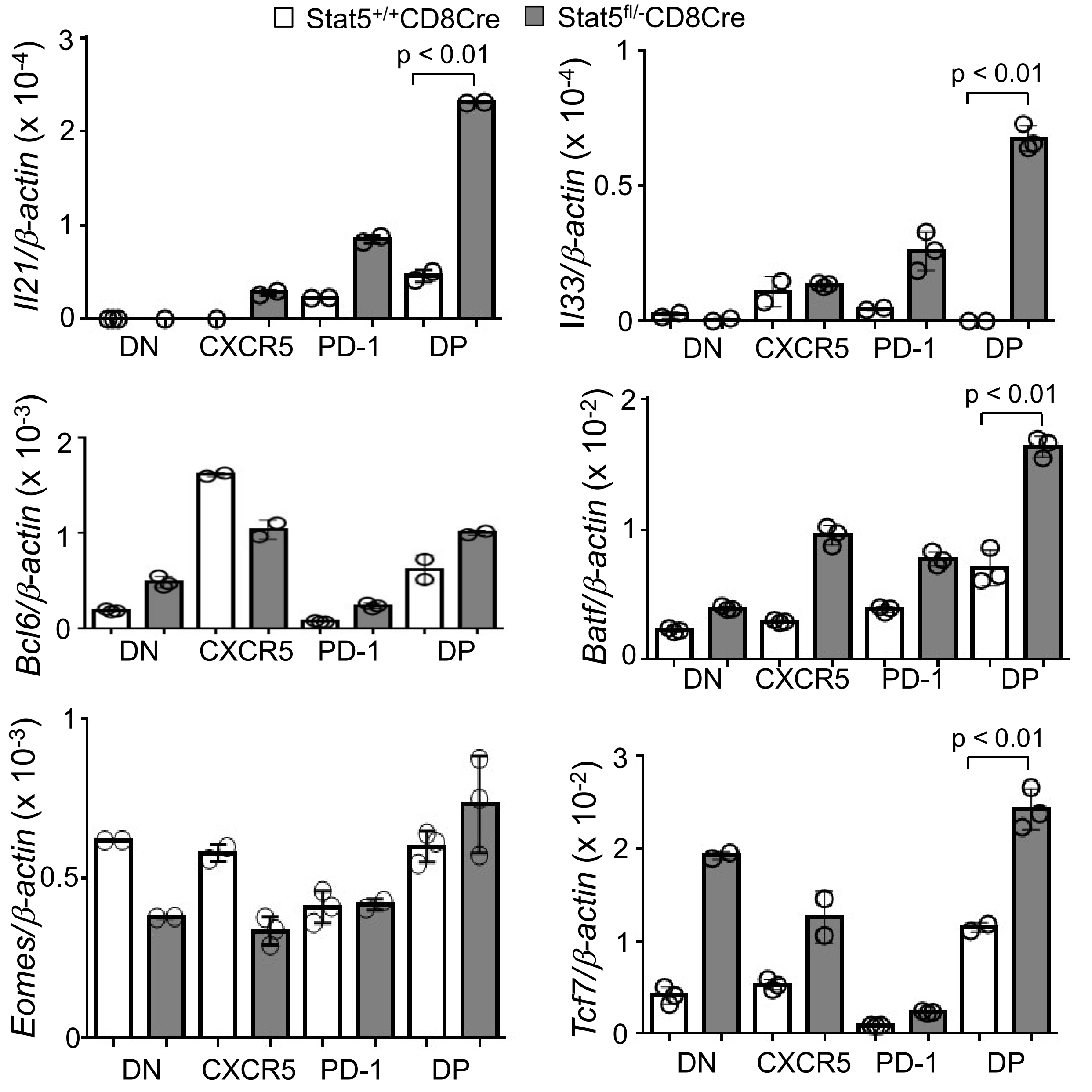

Supplement Fig 13. Confirmation of the up-regulated genes in Stat5-deficient CXCR5^{+}PD-1^{+}CD8^{+} T cells by RT-qPCR. Splenic CXCR5^{-}PD-1^{-}CD8^{+}(DN), CXCR5^{+}PD-1^{-}CD8^{+}(CXCR5), CXCR5^{-}PD-1^{+}CD8^{+}(PD-1) and CXCR5^{+}PD-1^{+}CD8^{+} (DP) T cells were sorted from Stat5$^{+/+}$CD8Cre and Stat5$^{fl/-}$CD8Cre mice 8 days after acute LCMV infection. Isolated mRNAs were subjected to RT-qPCR. Bar graphs show the mRNA levels of the indicated genes normalized to *β-actin* expression. Mean ± SD is shown. P-values were calculated with the unpaired two-tailed Student's *t*-test. The data shown are representative of 2 independent experiments.

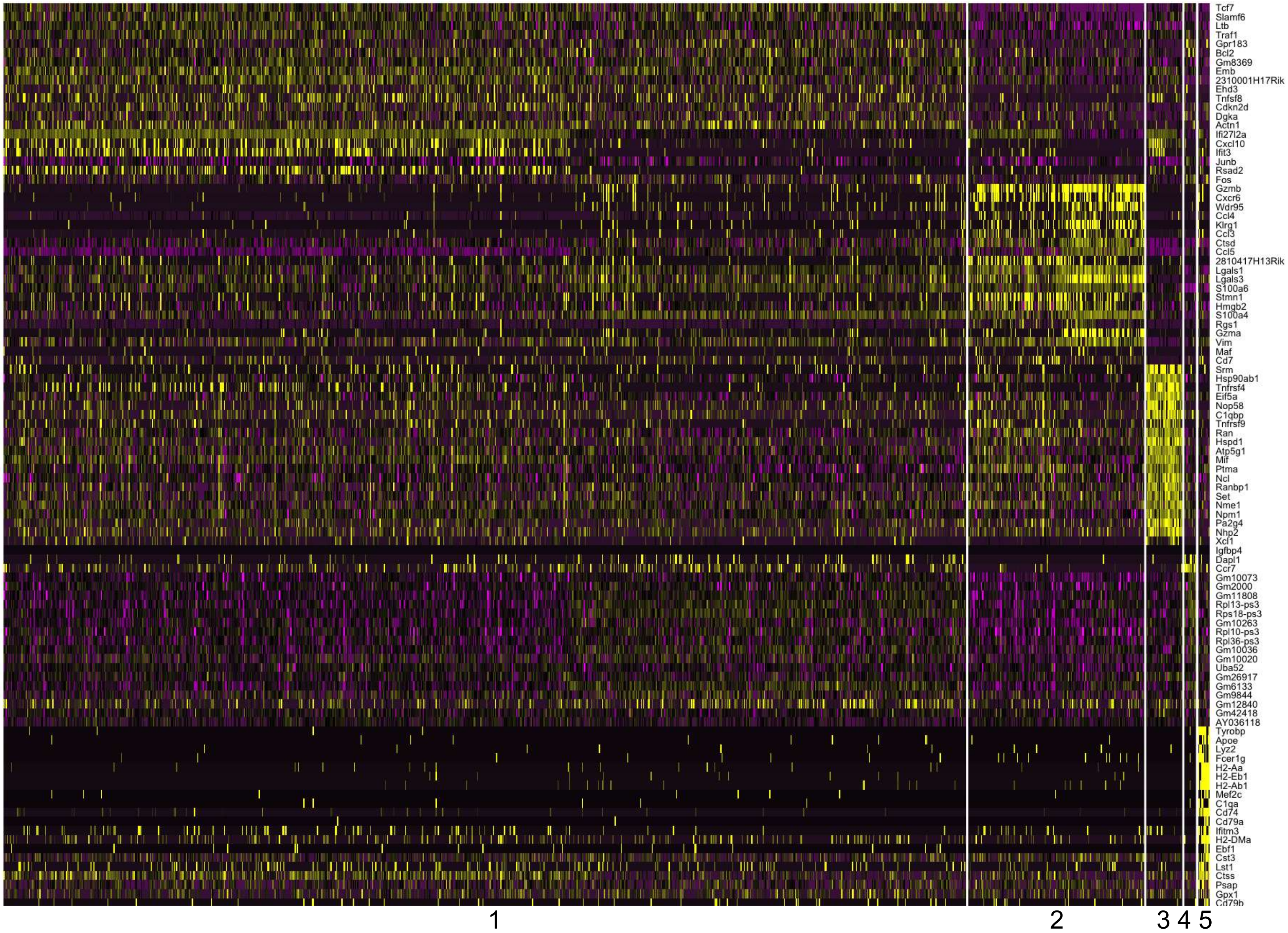

Supplementary Figure 14. Heatmap of genes high differentially expressed in each cluster of 5 cell clusters identified in CXCR5^{+}PD-1^{+}CD8^{+} T cells from Stat5$^{+/+}$CD8Cre/YFP or Stat5$^{fl/-}$CD8Cre/YFP mice by scRNA seq.

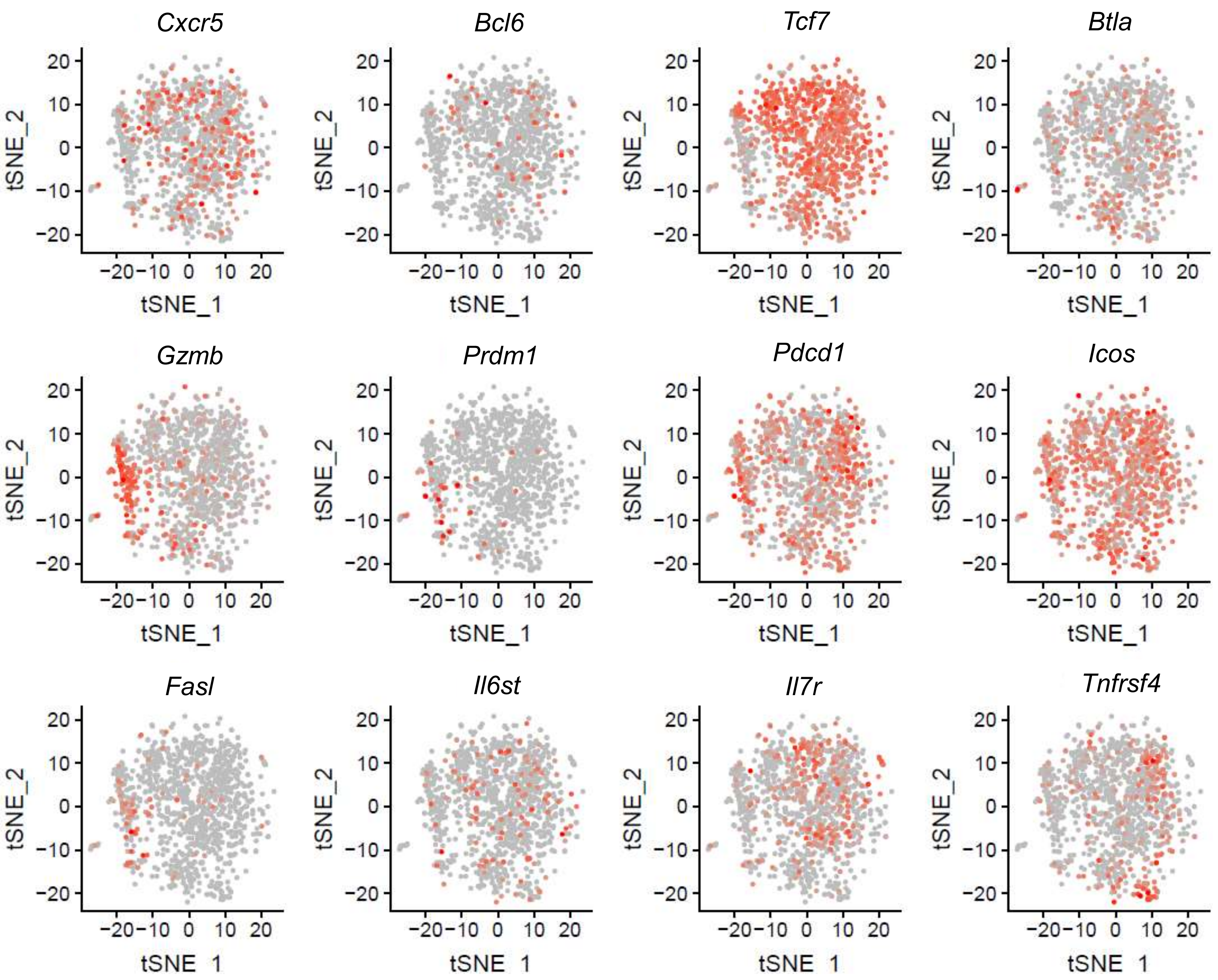

Supplementary Figure 15. Expression of the Tfh- and effector-related genes in CXCR5$^+$PD-1$^+$CD8$^+$ T cells. t-SNE plots show the expression of the Tfh- and effector-related genes in CXCR5$^+$PD-1$^+$CD8$^+$ T cells from acute LCMV-infected Stat5$^{+/+}$CD8Cre/YFP and Stat5$^{fl/-}$CD8Cre/YFP mice.

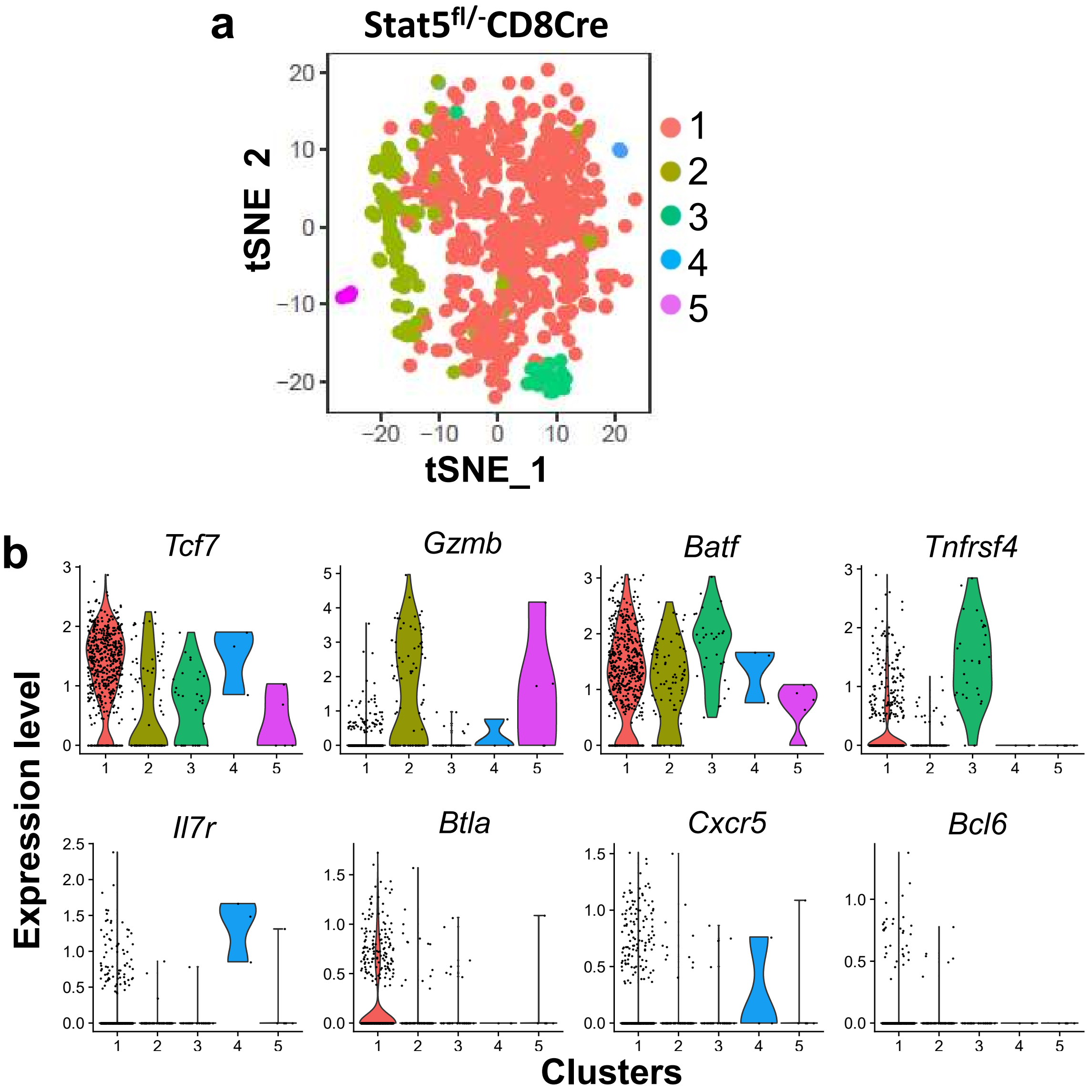

Supplementary Figure 16. a t-SNE plot of CXCR5$^+$PD-1$^+$CD8$^+$ cells from Stat5$^{fl/-}$CD8CreYFP mice. **b** Violin plots show the distribution of the expression level of *Tcf7, Gzmb, Batf, Tnfrsf4, Il7r, Btla, Bcl6* and *Cxcr5* in each cluster of CXCR5$^+$PD-1$^+$CD8$^+$ cells from Stat5$^{fl/-}$CD8CreYFP mice.

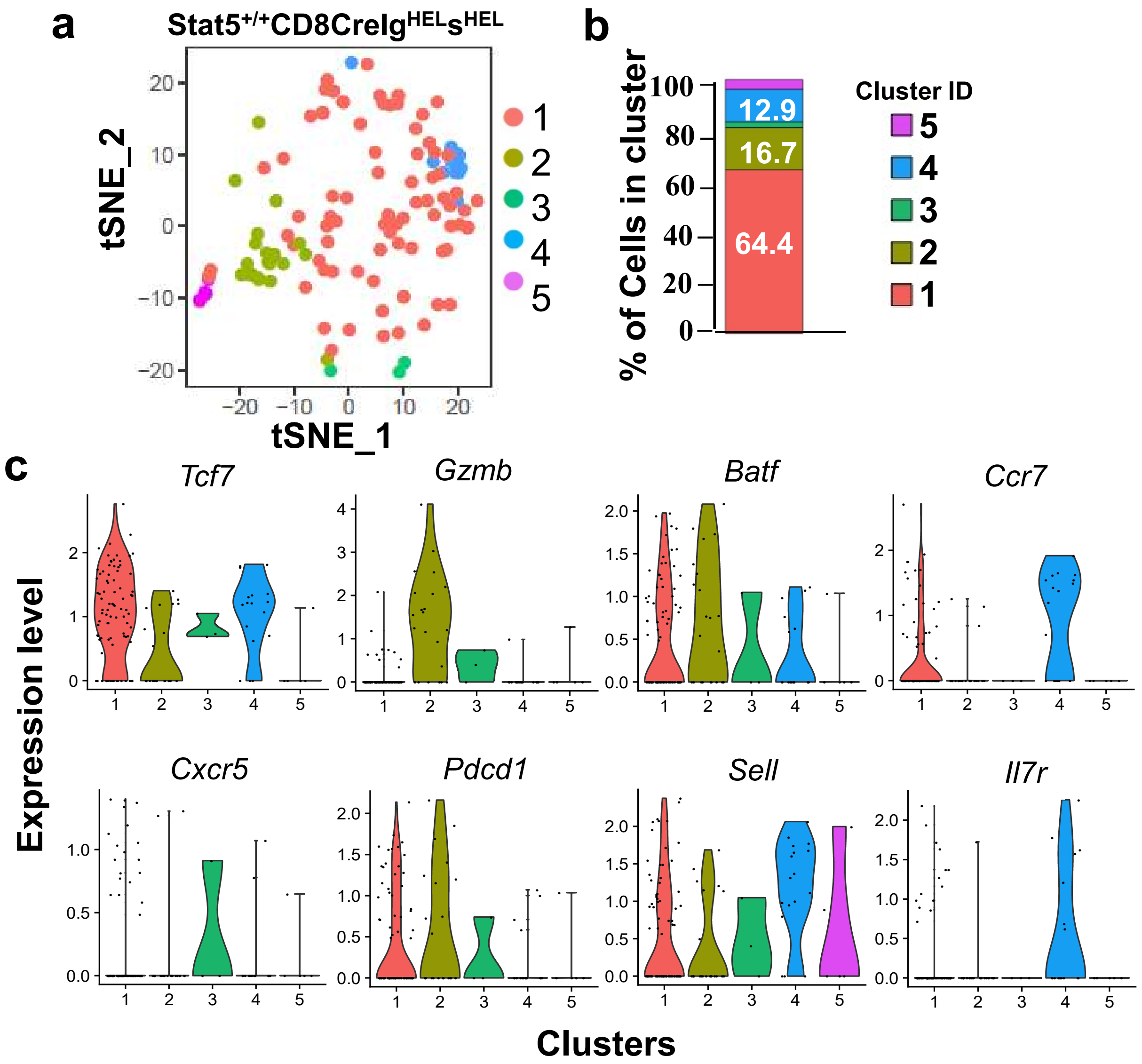

Supplementary Figure 17. **a** t-SNE plot of $CXCR5^+PD\text{-}1^+CD8^+$ T cells from $Stat5^{+/+}CD8Cre/YFPIg^{HEL}sHEL$ mice. **b** Bar graph indicates the percentages of cells in each cluster within $CXCR5^+PD\text{-}1^+CD8^+$ T cells. **c** Violin plots show the distribution of the expression level of *Tcf7, Gzmb, Batf, Ccr7, Cxcr5, Pdcd1, Sell* and *Il7r* in each cluster of $CXCR5^+PD\text{-}1^+CD8^+$ T cells from $Stat5^{+/+}CD8Cre/YFPIg^{HEL}sHEL$ mice.

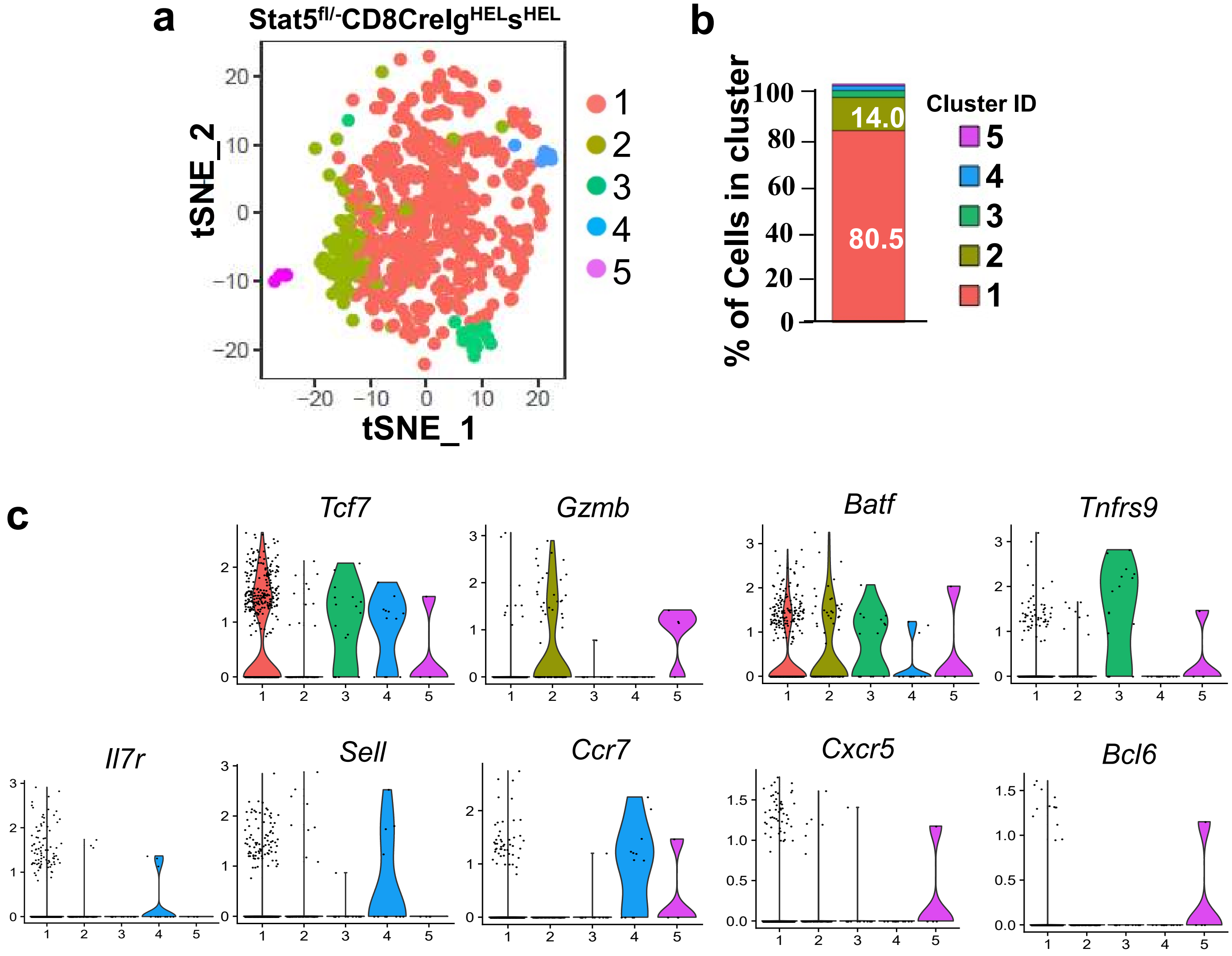

Supplementary Figure 18. **a** t-SNE plot of CXCR5$^+$PD-1$^+$CD8$^+$ T cells from Stat5$^{fl/-}$CD8Cre/YFPIg^{HEL}sHEL mice. **b** Bar graph indicates the percentages of cells in each cluster within CXCR5$^+$PD-1$^+$CD8$^+$ T cells. **c** Violin plots showing the distribution of the expression level of *Tcf7, Gzmb, Batf, Tnfrsf9, Il7r, Sell, Ccr7, Cxcr5* and *Bcl6* in each cluster of CXCR5$^+$ PD-1$^+$CD8$^+$ T cells of Stat5$^{fl/-}$CD8Cre/YFPIg^{HEL}sHEL mice.

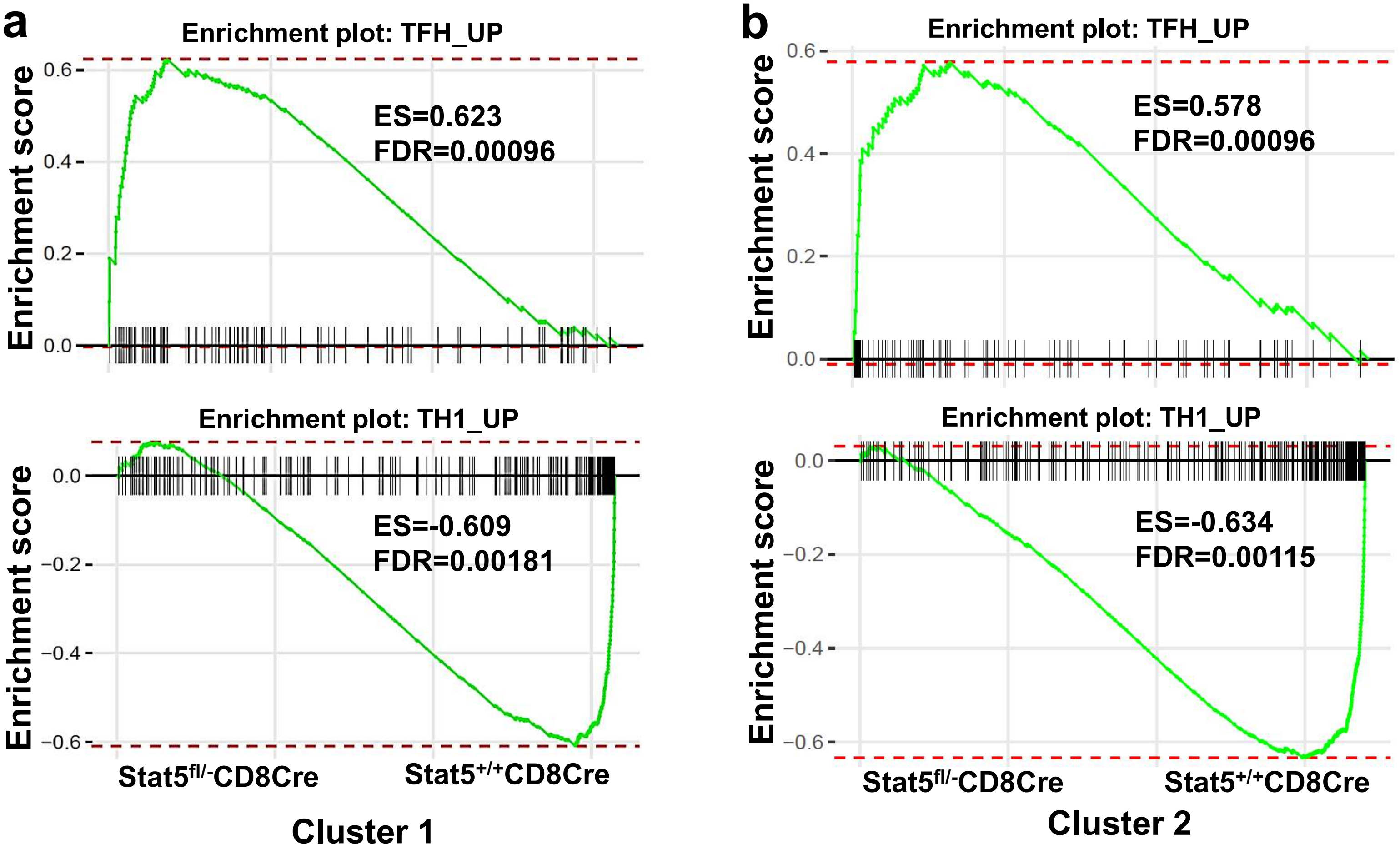

Supplementary Figure 19. Comparative GSEA of the Tfh and Th1 signatures in the cluster 1 and cluster 2 of wild-type and Stat5-deficient CXCR5^{+}PD-1^{+}CD8^{+} T cells. GSEA of the Tfh and Th1 signatures (GEO accession code GSE67334) in cluster 1 (**a**) and cluster 2 (**b**).

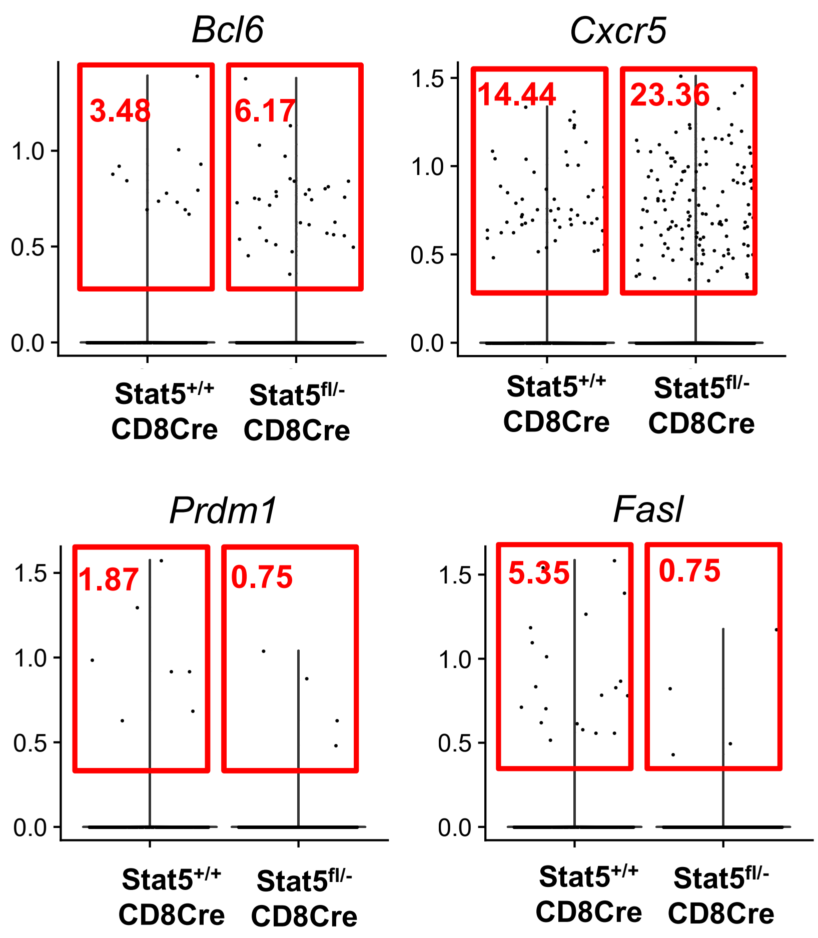

Supplementary Figure 20. Violin plots show the distribution of the expression level of *Bcl6, Cxcr5, Prdm1* and *Fasl* in the cluster 1 of CXCR5$^+$PD-1$^+$CD8$^+$ T cells from Stat5$^{+/+}$ CD8Cre/YFP and Stat5$^{fl/-}$CD8Cre/YFP mice. The number showed the percentages of indicated cells within the cluster 1.

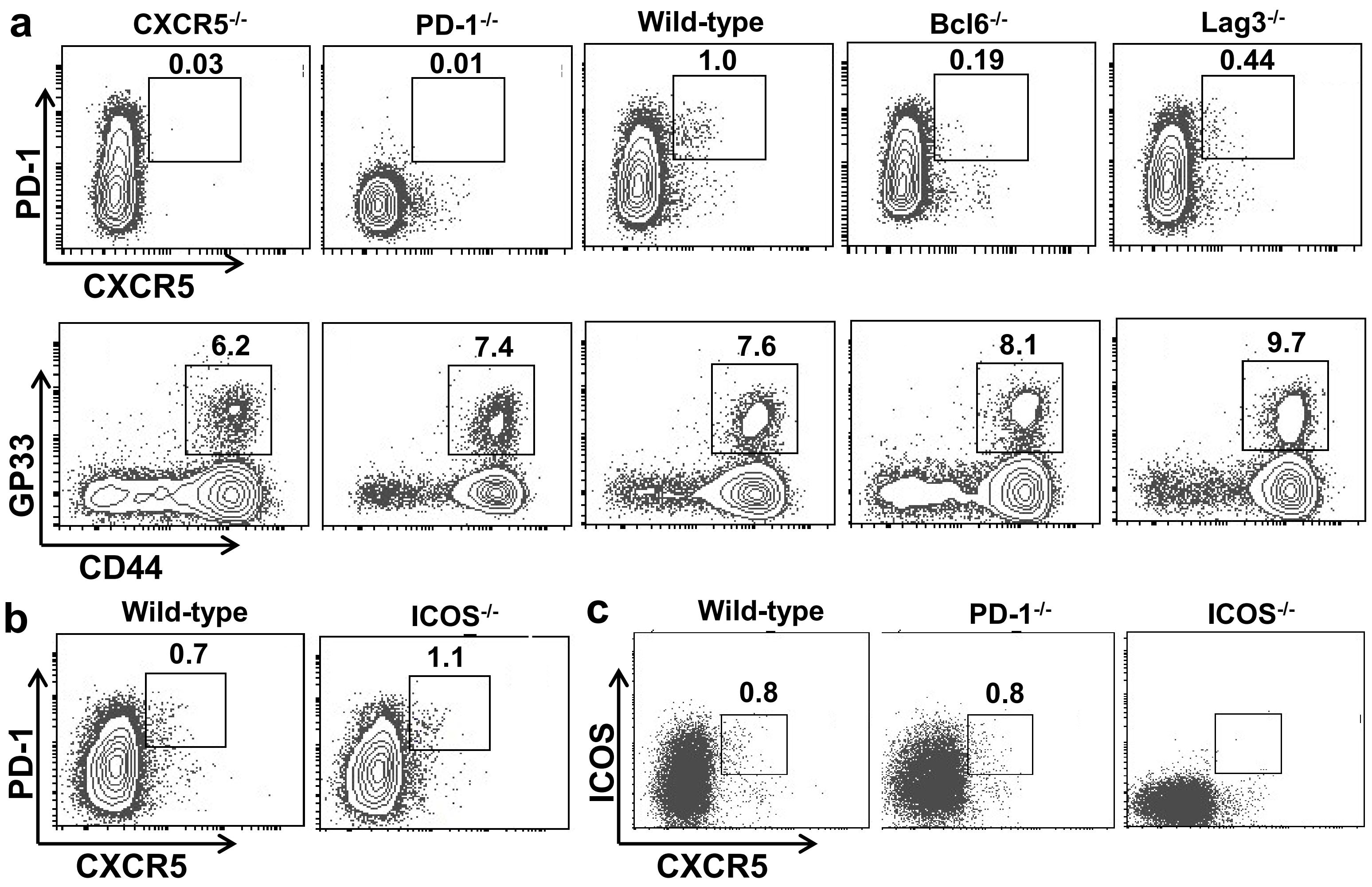

Supplementary Figure 21. Bcl6 or Lag3 deficiency impairs CXCR5$^+$PD-1$^+$CD8$^+$ T cell generation following acute LCMV infection. a Wild-type, Bcl6-, Lag3-, CXCR5-, PD-1- and ICOS-deficient mice were infected with LCMV (Armstrong). Eight days after infection, splenocytes were stained with anti-CD4, anti-CD8, anti-CXCR5 and anti-PD-1 antibodies (**a**,**b**), anti-CD8 and anti-CD44 antibodies and GP33-Tetramer (**a**), or anti-CD4, anti-CD8, anti-CXCR5 and anti-ICOS antibodies (**c**). Numbers indicate percentages of CXCR5$^+$PD-1$^+$, CD44$^+$GP33$^+$ or CXCR5$^+$ICOS$^+$ cells in the gated CD8$^+$ population. Data shown are representative of 2 independent experiments.

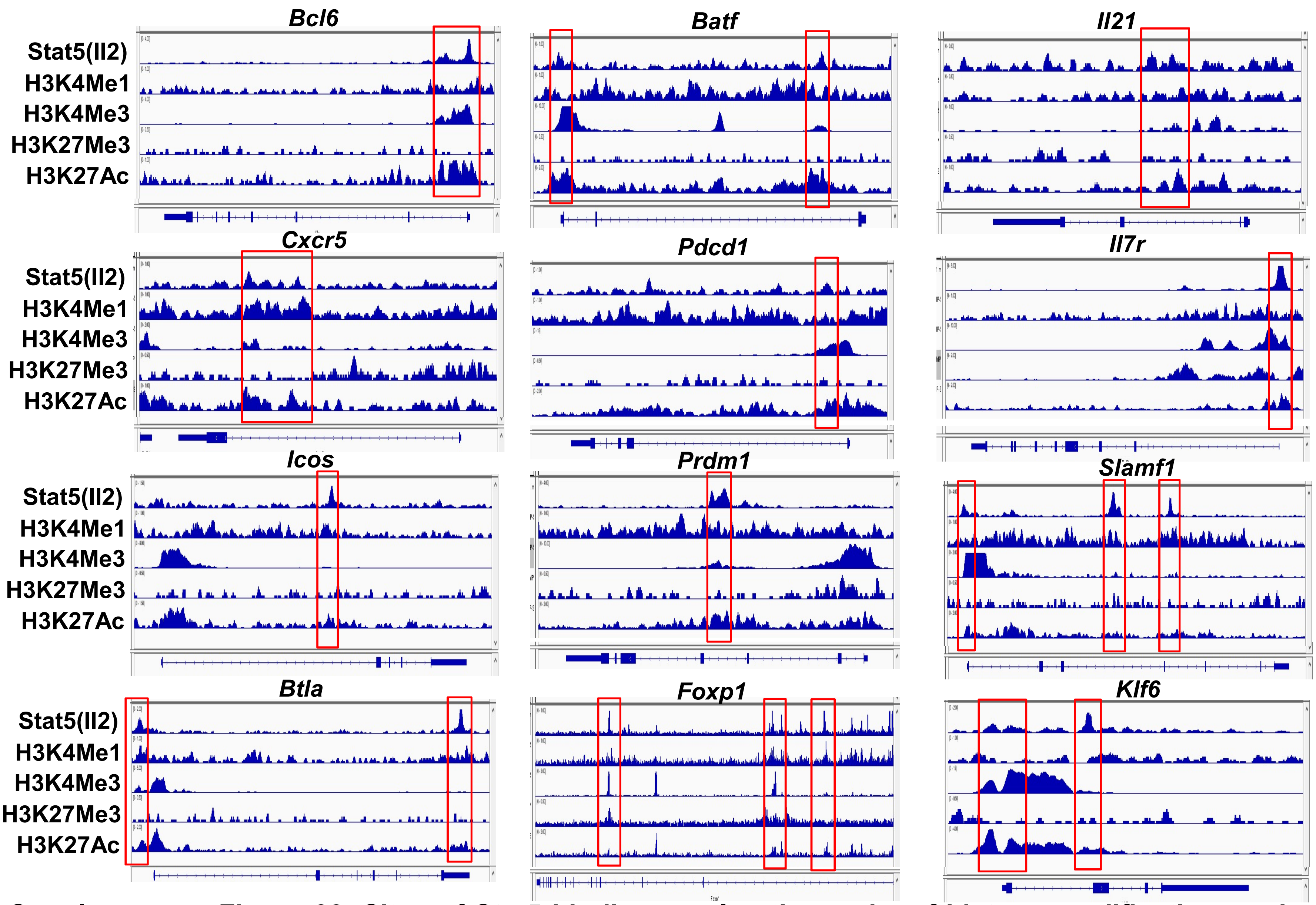

Supplementary Figure 22. Sites of Stat5 binding overlap the peaks of histone modification marks within the Tfh-related genes . ChIP-Seq QC Alignment (Bowtie2) analysis shows Stat5 DNA binding and histone modification sites at the Tfh-related gene loci in IL-2-activated CD8^{+} T cells and CD8^{+} T cells from acute LCMV-infected wild-type mice, respectively. Red frames indicate the examples of the overlapped regions of Stat5 binding sites and histone markers. Data from GSE72565 and GSE81888 were analyzed.

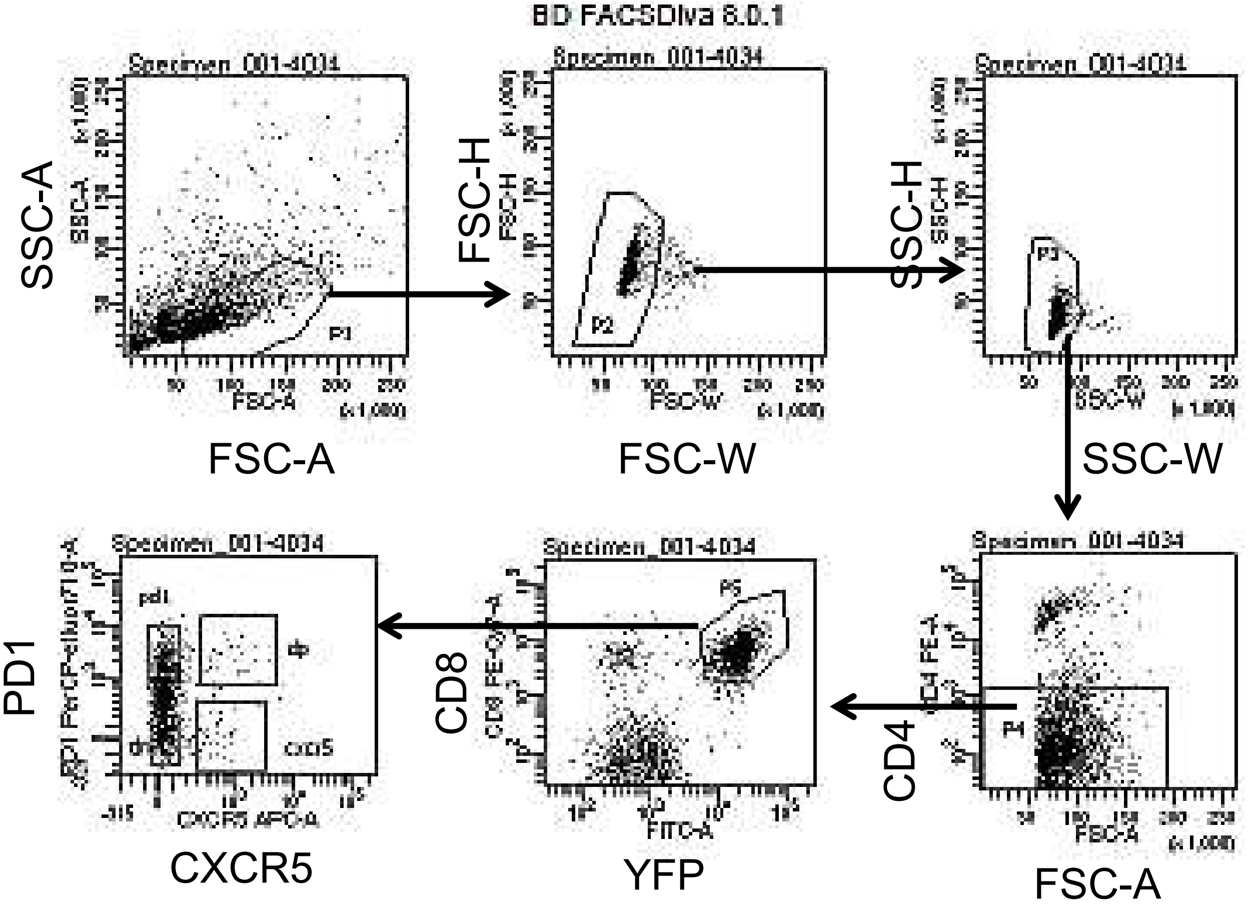

Supplementary Figure 23. The gating strategies for sorting CXCR5$^+$PD-1$^+$CD8$^+$ T cells. Splenocytes from LCMV-infected Stat5$^{+/+}$CD8Cre/YFP and Stat5$^{fl/-}$CD8Cre/YFP mice or Stat5$^{+/+}$ CD8Cre/YFPIg^{HEL}sHEL and Stat5$^{fl/-}$CD8Cre/YFPIg^{HEL}sHEL mice were stained with abti-CD4, anti-CD8, anti-CXCR5 and anti-PD-1 antibodies. The gating strategies for CXCR5$^-$PD-1$^-$CD8$^+$, CXCR5$^-$PD-1$^+$CD8$^+$, CXCR5$^+$PD-1$^-$CD8$^+$ and CXCR5$^+$PD-1$^+$CD8$^+$ T cells are shown.

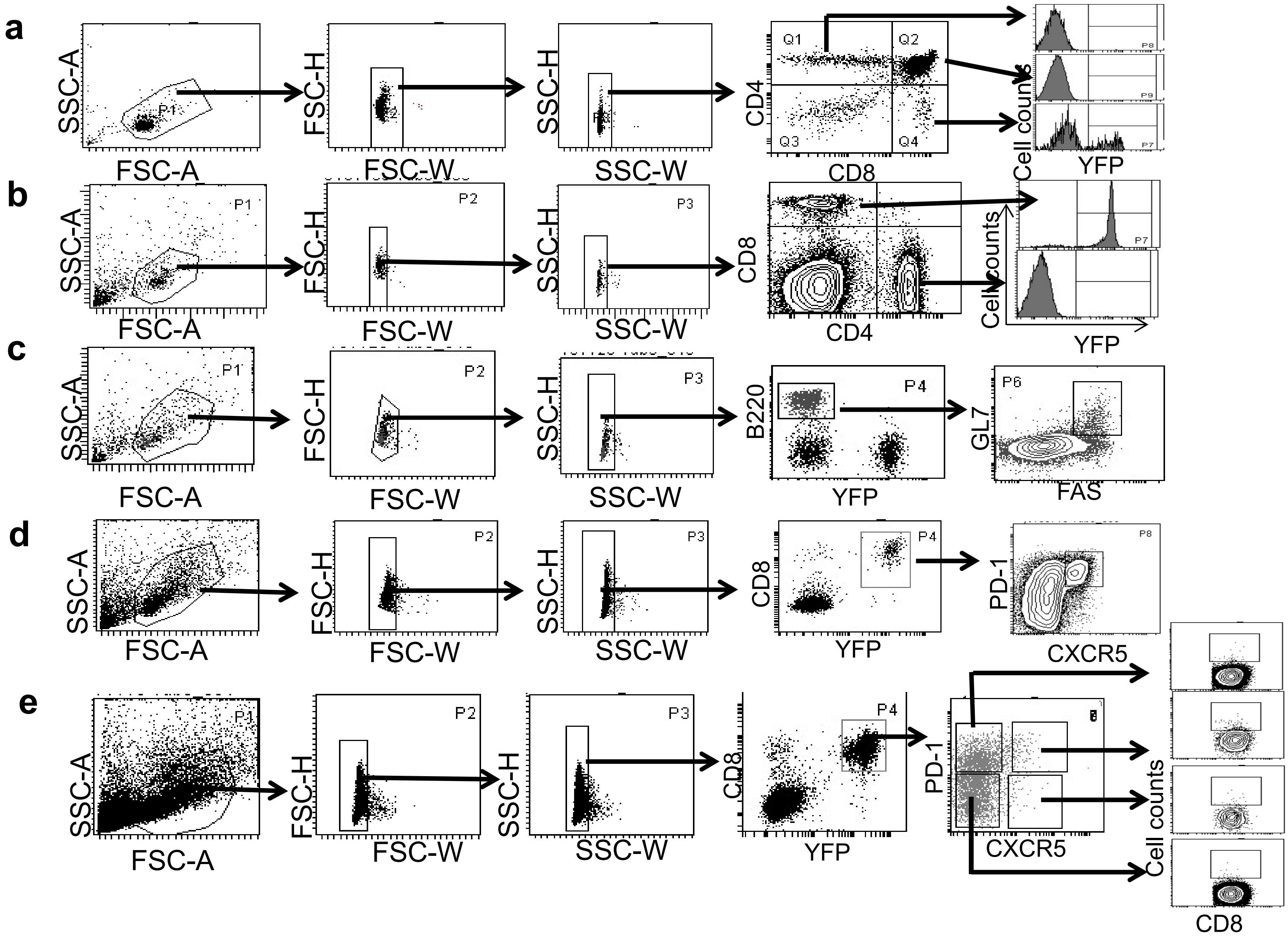

Supplementary Figure 24. Gating strategies for the FACS data. (a) Gating strategies for Fig. 1b, c. (b) Gating strategies for Fig. 1d, e. (c) Gating strategies for Fig. 3b. (d) Gating strategies for Fig. 3a, c. (e) Gating strategies for Fig. 7a, b.

Gene	Promoter	Genebody	Intergenic	Gene	Promoter	Genebody	Intergenic
Il21			x	*Nfatc1*		x	x
Bcl6	x	x		*Cxcr5*			x
Tcf-7			x	*Sh2d1a*			x
Lef-1		x	x	*Slamf1*	x	x	x
Tcf-4		x		*Tnfrsf18*			x
Batf			x	*Cd200*		x	
Lag3			x	*Foxo1*	x	x	x
Pdcd1	x		x	*Foxp1*	x	x	x
Icos	x	x	x	*Id3*			x
Tnfrsf4			x	*Prdm1*		x	x
Btla	x	x	x	*Ctla4*	x		x
Id2	x		x	*Il2rb*	x		x
KLF2			x	*Il7r*	x		x
Il2ra	x	x	x	*Il6ra*		x	x
Stat5		x		*Il6*			
Cd28	x		x	*Ifnb*			
Il27			x	*E2a*			
Il12			x	*Maf*			
Tgfb	x			*Stat3*			
Ifna			x	*Il2*			
Ifng	x		x	*Il7*			
IRF4	x		x	*Rc3h1*			
Ascl2			x	*Rc3h2*			
Jun	x		x				

Supplement Table 1. IL-2-induced Stat5 DNA binding of the Tfh-related genes in CD8^{+} T cells. The Tfh-related genes with IL-2-induced Stat5 binding were identified by analyzing Stat5 Chip-seq data from GSE72565. Up- or down-regulated genes in Stat5-deficient relative to wild-type CXCR5^{+}PD-1^{+}CD8^{+} T cells based on RNA-seq data were marked in red or green, respectively.

Supplementary Table 2. Summary of recent publications that have identified the $CXCR5^+CD8^+$ T cell subsets with different functions.

Population	Function	Animal model	Reference
$CXCR5^+$	Function as effector cells to control viral replication	Chronic LCMV infection	He, R. et al. Nature **537**, 412-428, (2016).
$CXCR5^+$	Function as effector cells to eliminate LCMV-infected $CD4^+$ Tfh cells and B cells in the B cell follicles	Chronic LCMV infection	Leong, Y. A. et al. Nature immunology **17**, 1187-1196, (2016)
$CXCR5^+$ $PD1^+$	Function as precursors of exhausted $CD8^+$ T cells that sustain the viral-specific $CD8^+$ T cells during chronic infection	Chronic LCMV infection	Im, S. J. et al. Nature **537**, 417-421, (2016).
$CXCR5^+$	Function as B cell helpers	Runx3 KO mice Acute LCMV infection	Shan, Q. *et al.* Nat. Immunol. **18**, 931-939, (2017)
$TCF1^{high}$ $Tim3^{low}$ $Blimp1^{low}$ $CXCR5^+$	Transcriptionally resemble T_{FH} cells, function as progenitor cells that either remain as progenitors or terminally differentiate into $TCF1^{low}Tim3^{high}$ cells.	Chronic LCMV infection	Wu, T. *et al.* Sci. Immunol. **1**, doi:10.1126/sciimmunol.aai8593 (2016).
$CXCR5^+$ $IcosL^+$	Function as regulatory T cells that suppress $CD4^+$ Tfh cells and thus control self tolerance	Qa-1(D227K) KI mice Acute LCMV infection	Kim, H. J. *et al.* Nature **467**, 328-332, (2010).
$CXCR5^+$ $PD1^+$	Function as Tfh cells to breakdown of B-cell tolerance	Stat5-deficient mice Acute LCMV infection	Current study

Supplementary Table 3. Antibodies for flow cytometry (IC), Western blot (WB) analysis and immunohistochemistry (IHC).

Epitope	Format	Cat#	Vender	Dilution
B220	Apc	17-0452	eBioscience	300 (IC)
CD4 ,	Apc	17-0041	eBioscience	300 (IC)
B220	PE-Cye7	25-0452	eBioscience	300 (IC)
CD8	PE-Cye7	25-0081	eBioscience	300 (IC)
CD40L	PE	12-1541	eBioscience	300 (IC)
PD-1	PerCP-efluor 710	46-9985	eBioscience	200 (IC)
Fas	PE	554258	BD Biosciences Pharmingen.	300 (IC)
IgG1	Apc	550874	BD Biosciences Pharmingen.	300 (IC)
GL7	Alexa Fluor 647	551529	BD Biosciences Pharmingen.	300 (IC)
CD4	Apc-Cy7	552051	BD Biosciences Pharmingen.	300 (IC)
CD44	Apc-Cy7	103028	BD Biosciences Pharmingen.	300 (IC)
Streptavidin	Apc	016-130-084	Jackson ImmunoResearch Laboratories	500 (IC)
CD1d Tetramer	Apc	CD1dPBS-57	NIH Tetramer Core Facility	100 (IC)
CXCR5	Biotin	2024-01	eBioscience	100 (IC)
Bcl6	Alexa Fluor 647	561525	BD Biosciences Pharmingen.	300 (IC)
IFNg	PE-Cy7	505826	Biolegend	300 (IC)
TCF1		2203	Cell Signaling	300 (IC)
Klrg1	PE-Cy7	25-5893	eBioscience	300 (IC)
Icos	PE	12-9942-82	eBioscience	300 (IC)
Tim3	PE	566348	BD Biosciences Pharmingen.	300 (IC)
BTLA	PE	12-5950-82	eBioscience	400 (IC)
BATF		8638	Cell Signaling	300 (IC)
STAT5	for WB	S2150	Transduction Laboratory	1000 (WB)
b-Actin	for WB	MAB1501R	Millpore	5000 (WB)
B220	PE-CF594	562313	BD Biosciences Pharmingen.	100 (IHC)
GL7	eFluor450	48-5902-80	eBioscience	75 (IHC)
B220	eFluor450	48-0452-82	eBioscience	100 (IHC)
CXCR5	Apc	145505	Biolegend	75 (IHC)
GL7	Alexa Fluor 647	551529	BD Biosciences Pharmingen.	75 (IHC)
