# Supplementary material for: CXCR5+PD-1+ follicular helper CD8 T cells control B cell tolerance
Source: Nat Commun. 2019 Sep 27;10:4415. doi: 10.1038/s41467-019-12446-5 (PMC6765049; doi:10.1038/s41467-019-12446-5)
Supplement: Supplementary file 3 — Reporting Summary [file 41467_2019_12446_MOESM3_ESM.pdf]

nature research

Corresponding author(s): Demin Wang
Last updated by author(s): 2019-9-1

Reporting Summary

Nature Research wishes to improve the reproducibility of the work that we publish. This form provides structure for consistency and transparency in reporting. For further information on Nature Research policies, see Authors & Referees and the Editorial Policy Checklist.

Statistics

For all statistical analyses, confirm that the following items are present in the figure legend, table legend, main text, or Methods section.

n/a	Confirmed	
☐	☒	The exact sample size (*n*) for each experimental group/condition, given as a discrete number and unit of measurement
☐	☒	A statement on whether measurements were taken from distinct samples or whether the same sample was measured repeatedly
☐	☒	The statistical test(s) used AND whether they are one- or two-sided<br>*Only common tests should be described solely by name; describe more complex techniques in the Methods section.*
☒	☐	A description of all covariates tested
☒	☐	A description of any assumptions or corrections, such as tests of normality and adjustment for multiple comparisons
☐	☒	A full description of the statistical parameters including central tendency (e.g. means) or other basic estimates (e.g. regression coefficient) AND variation (e.g. standard deviation) or associated estimates of uncertainty (e.g. confidence intervals)
☐	☒	For null hypothesis testing, the test statistic (e.g. *F*, *t*, *r*) with confidence intervals, effect sizes, degrees of freedom and *P* value noted<br>*Give P values as exact values whenever suitable.*
☒	☐	For Bayesian analysis, information on the choice of priors and Markov chain Monte Carlo settings
☒	☐	For hierarchical and complex designs, identification of the appropriate level for tests and full reporting of outcomes
☒	☐	Estimates of effect sizes (e.g. Cohen's *d*, Pearson's *r*), indicating how they were calculated

Our web collection on statistics for biologists contains articles on many of the points above.

Software and code

Policy information about availability of computer code

Data collection	All instruments, computer code and software used to collect data were described in details in the Methods section.
Data analysis	All computer code and software for data analysis were described in details in the Methods section.

For manuscripts utilizing custom algorithms or software that are central to the research but not yet described in published literature, software must be made available to editors/reviewers. We strongly encourage code deposition in a community repository (e.g. GitHub). See the Nature Research guidelines for submitting code & software for further information.

Data

Policy information about availability of data

All manuscripts must include a data availability statement. This statement should provide the following information, where applicable:

- Accession codes, unique identifiers, or web links for publicly available datasets
- A list of figures that have associated raw data
- A description of any restrictions on data availability

The authors declare that the data supporting the findings are available in the article, the supplementary information, the Source data file and the NCBI Gene Expression Ominibus (GEO), GSE136642.

Field-specific reporting

Please select the one below that is the best fit for your research. If you are not sure, read the appropriate sections before making your selection.

☒ Life sciences ☐ Behavioural & social sciences ☐ Ecological, evolutionary & environmental sciences

Life sciences study design

All studies must disclose on these points even when the disclosure is negative.

Sample size	Sample sizes were determined based on the principle of using the minimum number of animals to provide adequate statistical power.
Data exclusions	None
Replication	All experiments are representative of at least two independent experiments.
Randomization	Experimental mice were randomly allocated.
Blinding	None

Reporting for specific materials, systems and methods

We require information from authors about some types of materials, experimental systems and methods used in many studies. Here, indicate whether each material, system or method listed is relevant to your study. If you are not sure if a list item applies to your research, read the appropriate section before selecting a response.

Materials & experimental systems

n/a	Involved in the study	
☐	☒	Antibodies
☒	☐	Eukaryotic cell lines
☒	☐	Palaeontology
☐	☒	Animals and other organisms
☒	☐	Human research participants
☒	☐	Clinical data

Methods

n/a	Involved in the study	
☒	☐	ChIP-seq
☐	☒	Flow cytometry
☒	☐	MRI-based neuroimaging

Antibodies

Antibodies used	The information for all antibodies is included in the Methods section and the Supplementary Information.
Validation	All antibodies were validated by the manufacturers.

Animals and other organisms

Policy information about studies involving animals; ARRIVE guidelines recommended for reporting animal research

Laboratory animals	CD8CreStat5+/+, CD8CreStat5+/-, CD8CreStat5fl/fl mice, IgHEL transgenic (C57BL/6, MD4), sHEL transgenic (C57BL/6, ML5), BM1, Rag-1-deficient, CD45.1 congenic mice, CXCR5-deficient, PD-1-deficient, Lag3-deficient and CD40-deficient mice, and CD4CreBcl6fl/fl mice were used.
Wild animals	None
Field-collected samples	None
Ethics oversight	The animal experiments were performed using protocols approved by the Institute Animal Care and Use Committee at the Medical College of Wisconsin.

Note that full information on the approval of the study protocol must also be provided in the manuscript.

Flow Cytometry

Plots

Confirm that:

☒ The axis labels state the marker and fluorochrome used (e.g. CD4-FITC).

☒ The axis scales are clearly visible. Include numbers along axes only for bottom left plot of group (a 'group' is an analysis of identical markers).

☒ All plots are contour plots with outliers or pseudocolor plots.

☒ A numerical value for number of cells or percentage (with statistics) is provided.

Methodology

Sample preparation	Single-cell suspensions of mouse thymus, spleen and bone marrow cells were treated with Gey's solution to remove RBC and then stained with a combination of fluorescence-conjugated antibodies.
Instrument	LSR II (Becton Dickinson)
Software	FACSDiva
Cell population abundance	The purity of cells sorted for sequencing was greater than 99% as examined by a post-sort FACS analysis.
Gating strategy	Gating strategy for all flow cytometry experiments is included in the Supplementary Information.

☒ Tick this box to confirm that a figure exemplifying the gating strategy is provided in the Supplementary Information.
